# New 1-(3-Nitrophenyl)-5,6-dihydro-4*H*-[1,2,4]triazolo[4,3-*a*][1,5]benzodiazepines: Synthesis and Computational Study

**DOI:** 10.3390/molecules20045392

**Published:** 2015-03-26

**Authors:** Lidija Kosychova, Antanas Karalius, Zita Staniulytė, Romualdas Aleksas Sirutkaitis, Algirdas Palaima, Audrius Laurynėnas, Žilvinas Anusevičius

**Affiliations:** 1 Institute of Biochemistry, Vilnius University, Mokslininku 12, Vilnius LT-08662, Lithuania; E-Mails: zita.staniulyte@bchi.vu.lt (Z.S.); romualdas.sirutkaitis@bchi.vu.lt (R.A.S.); algirdas.palaima@bchi.vu.lt (A.P.); audrius.laurynenas@gmail.com (A.L.); zilvinas.anusevicius@bchi.vu.lt (Z.A.); 2 Department of Technological Processes, Klaipeda University, H. Manto 84, Klaipeda LT-91001, Lithuania; 3 Royal Institute of Technology, Department of Chemistry, Teknikringen 36, Stockholm S-10044, Sweden; E-Mail: antanas.karalius@gmail.com

**Keywords:** [1,2,4]triazolo[4,3-*a*][1,5]-benzodiazepine, 3-nitrobenzohydrazide, five-membered fused heterocycles, quantum mechanical calculation, density functional theory (DFT), DFT global descriptors, DFT local descriptors, Fukui index

## Abstract

Triazole derivatives constitute an important group of heterocyclic compounds have have been the subject of extensive study in the recent past. These compounds have shown a wide range of biological and pharmacological activities. In this work, new fused tricyclic 1-(3-nitrophenyl)-5,6-dihydro-4*H*-[1,2,4]triazolo[4,3-*a*][1,5]-benzodiazepines have been synthesized by the thermal cyclization of *N*'-(2,3-dihydro-1*H*-1,5-benzodiazepin-4-yl)-3-nitrobenzohydrazides. After screening ethanol, toluene and 1-butanol as solvents, butanol-1 was found to be the best choice for the cyclization reaction in order to obtain the highest yields of tricyclic derivatives. The chemical structures of the synthesized compounds were elucidated by the analysis of their IR, ^1^H- and ^13^C-NMR spectral data. For tentative rationalization of the reaction processes, the global and local reactivity indices of certain compounds, taking part in the reaction pathway, were assessed by means of quantum mechanical calculations using the conceptual density functional theory (DFT) approach. This work could be useful for the synthesis of new heterocyclic compounds bearing a fused triazole ring.

## 1. Introduction

Our literature survey revealed that triazole derivatives belonging to an important group of heterocyclic compounds have been the subject of extensive study in the recent past. The 1,2,4-triazole nucleus has been incorporated into a wide variety of therapeutically interesting compounds associated with diverse antibacterial, antifungal, anti-inflammatory and antihypertensive activities [1,2,3,4,5,6]. Fused heterocyclic triazoles also possess important clinical applications. Several compounds containing 1,2,4-triazole rings are well known as non-steroidal compounds used for treatment of cancer, some of them showing an anticonvulsant, antitumor and antiviral activity [7,8,9,10,11]. The chemistry of 1,5-benzodiazepines and their fused heterocyclic derivatives has received considerable attention owing to their synthetic importance and a broad spectrum of useful properties. It is known that the activity of fused [1,2,4]triazolo[4,3-*a*][1,4]benzodiazepines could be qualitatively modified by substitution at C-1 atom [12]. Among the various 1,2,4-triazoles molecules designed and synthesized in recent years, nitro-substituted arene 1,2,4-triazole derivatives possess biological activities as well as high energetic and anti-corrosion qualities [13,14,15,16].

Keeping these observations in mind and in continuation of our research on the synthesis of different nitrogen-containing heterocyclic rings, annulated onto the basic 1,5-benzodiazepine bicyclic system [17,18,19,20,21,22], in this work, we have carried out the synthesis of some new 1-(3-nitrophenyl)-5,6-dihydro-4*H*-[1,2,4]triazolo[4,3-*a*][1,5]benzodiazepine derivatives. For tentative rationalization of the reaction outcome, the global and local reactivity indices of certain compounds taking part in the synthesis pathway were assessed by means of the B3LYP functional method within a framework of the conceptual density functional theory (DFT) approach.

## 2. Results and Discussion

### 2.1. Chemistry

**Scheme 1:**
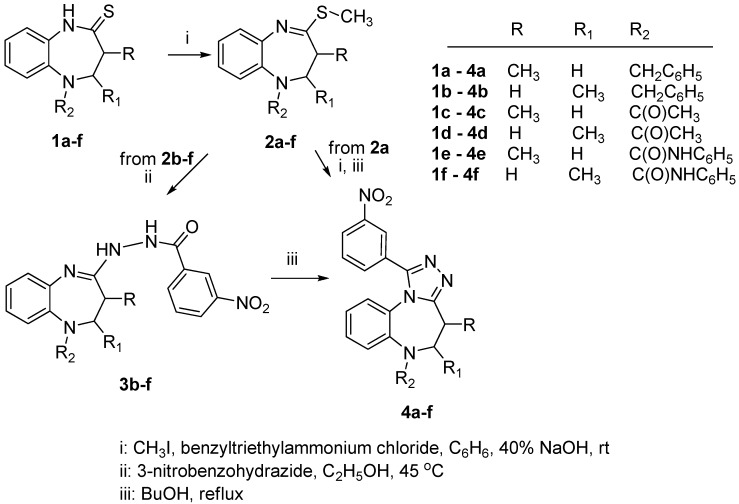
Synthesis of 1-(3-nitrophenyl)-5,6-dihydro-4*H*-[1,2,4]triazolo[4,3-*a*][1,5]benzodiazepines.

The synthesis pathway for constructing 1-(3-nitrophenyl)-5,6-dihydro-4*H*-[1,2,4]triazolo[4,3-*a*][1,5]benzodiazepines **4a**–**f** is shown in Scheme 1. The precursors of thiolactams **1a**–**f** were previously described by us [22,23,24]. Compounds 1a–f were converted into the thioethers **2a**–**f** by phase-transfer catalyzed alkylation with an excess of iodomethane. The thioethers were synthesized to avoid the lack of reactivity of the corresponding thiolactams toward nucleophiles. The compounds **2a**,**b**,**d** were reported previously [25,26], while compounds **2c**,**e**,**f** have now been obtained in good yields for the first time according to this procedure, The structures of the compounds were supported by the analysis of their IR, ^1^H- and ^13^C-NMR spectra. The IR spectra exhibited typical C=N stretching bands between 1598–1592 cm^−1^ as well as C=O stretching bands at 1679–1650 cm^−1^. In the ^1^H-NMR spectra, signals derived from the methylthio group were recorded as singlets at 2.40–2.48 ppm. This group resonated at 12.9–13.2 ppm in the ^13^C-NMR spectrum.

The reaction of thioethers **2a**–**f** with freshly recrystallized 3-nitrobenzohydrazide in anhydrous ethanol gave *N*'-(2,3-dihydro-1*H*-1,5-benzodiazepin-4-yl)-3-nitrobenzohydrazides **3a**–**f**. Monitoring by TLC, it has been found that the optimal reaction temperature was about 45 °C, and the reaction time was 18–26 h. Poorly soluble non-cyclic products **3a**–**f** formed a precipitate, whereas the excess or unreacted starting material remained in solution. The TLC monitoring showed that under these conditions only one new product was obtained. Compounds **3b**–**f** were isolated as crystalline substances and fully identified. As reaction product **3a** decomposed or partial cyclized the crude material was used as for further cyclization.

The structure of compounds **3b**–**f** was confirmed on the basis of their spectroscopic characteristics. The IR spectra showed the appearance of the NO_2_ vibration in 1531–1528 cm^−1^ and 1352–1347 cm^−1^. The IR spectrum of **3b** exhibited a sharp peak at 1671 cm^−1^ due to the C=O absorption. Meanwhile, in the ^1^H-NMR spectra of **3b**–**d**, **f**, the signals derived from the NH groups were recorded as singlet at 8.85–8.98 and 10.54–10.64 ppm and increased the number of protons in the aromatic region.

The thermal condensation of compounds **3a**–**f** was performed after a number of experiments in order to select the best conditions for the cyclization reactions to obtain the highest yields of the expected tricyclic derivatives. Suspensions of *N*'-(2,3-dihydro-1*H*-1,5-benzodiazepin-4-yl)-3-nitro-benzohydrazides **3a**–**f** were heated at reflux in different solvents. After screening ethanol, toluene and 1-butanol as solvents, it was found that the desired product **4e** was obtained only by refluxing in 1-butanol and that this solvent was thus the best choice for the reaction, because the reaction proceeded well in a high boiling solvent. The results are outlined in Table 1.

**Table 1:** Synthesis of 1-(3-nitrophenyl)-5,6-dihydro-4*H*-[1,2,4]triazolo[4,3-*a*][1,5]benzodiazepines **4a**–**f** in different solvents.

Compound	Ethanol, Time (h)	Toluene, Time (h)	1-Butanol, Time (h)
**4a**	36	19	9
**4b**	43	21	11
**4c**	41	20	14
**4d**	48	25	16
**4e**	–	‒	21
**4f**	47	22	15

Generally, it was demonstrated that cyclization of the compounds proceeded within different time periods for each compound and depended on its structure, as well as on the solubility in an appropriate solvent. The 1-(3-nitrophenyl)-5,6-dihydro-4*H*-[1,2,4]triazolo[4,3-*a*][1,5]benzodiazepines **4a**–**f** were obtained in good yields (65%–85%) and identified by their ^1^H spectra which shows the disappearance of the NH groups at 8.85–8.98 ppm and 10.54–10.64 ppm. The ^13^C-NMR resonances of the C-1 atom of the triazole ring were observed at 150.2–150.6 ppm. In addition, the IR spectra of compounds **4a** and **4b** displayed no signals originating from the CO group.

Recently we have described the preparation of substituted 5,6-dihydro-4*H*-[1,2,4]triazolo[4,3-*a*][1,5]benzodiazepine derivatives from the corresponding hydrazides [26]. It was found that the reaction of 1-acetyl-2-methyl-4-(methylsulfanyl)-2,3-dihydro-1*H*-1,5-benzodiazepine **2d** with benzo-hydrazide in anhydrous ethanol at room temperature gave *N*'-(1-acetyl-2-methyl-2,3-dihydro-1*H*-1,5-benzodiazepin-4-yl)benzohydrazide in good yield, and the cyclization of this compound by refluxing in anhydrous ethanol gave 6-acetyl-5-methyl-1-phenyl-5,6-dihydro-4*H*-[1,2,4]triazolo[4,3-*a*][1,5]-benzodiazepine. Similar reactions with 3-nitrobenzohydrazide which are outlined in Table 2 were found to occur only at higher temperature and much more slowly, implying that the presence of a nitro group on the aromatic ring deactivates 3-nitrobenzohydrazide as a nucleophile.

**Table 2:** Favorable reaction conditions for the synthesis of compounds.

Compound	Solvent	Temp (°C )	t (h)	Yield (%)
*N*'-(1-acetyl-2-methyl-2,3-dihydro-1*H*-1,5-benzodiazepin-4-yl)benzohydrazide	ethanol	room	16	91
*N*'-(1-acetyl-2-methyl-2,3-dihydro-1*H*-1,5-benzodiazepin-4-yl)-3-nitrobenzohydrazide	ethanol	45	18	65
6-acetyl-5-methyl-1-phenyl-5,6-dihydro-4 *H*-[1,2,4]triazolo[4,3-*a*][1,5]benzodiazepine	ethanol	78	8	77
6-acetyl-5-methyl-1-(3-nitrophenyl)-5,6-dihydro-4 *H*-[1,2,4]triazolo[4,3-*a*][1,5]benzodiazepine	1-butanol	118	20	85

### 2.2. Computational Study

To tentatively rationalize the interaction of 1-acetyl-2-methyl-4(methylsulfanyl)-2,3-dihydro-1*H*-1,5-benzodiazepine (thioether compound **2d**) with benzohydrazide and 3-nitrobenzohydrazide (Scheme 1, synthesis pathway ii), as well as to provide a partial explanation for the cyclization reactions of *N*'-(1-acetyl-2-methyl-2,3-dihydro-1*H*-1,5-benzodiazepin-4-yl)benzohydrazide and *N*'-(1-acetyl-2-methyl-2,3-dihydro-1*H*-1,5-benzodiazepin-4-yl)-3-nitrobenzohydrazide (Scheme 1, synthesis pathway iii), a quantum mechanical computation study of the compounds was performed within the framework of the density functional theory (DFT) approach ([27,28,29,30] and references therein). Within the DFT framework, the estimated global and local reactivity indices of the compounds, reflecting their electrophilic/nucleophilic powers, can be used to assess *a priori* the reactivity of chemical species involved in a chemical process at the ground state of reagents ([31], and references therein). The global electrophilic index (ϖ) of the molecules, representing their global electrophilic power, is defined using the following expression, ϖ = µ^2^/(2η) [28], where µ and η are the electronic chemical potential and the chemical hardness of the compounds, respectively. In accordance with Koopmans’ approximation for the closed shell-molecules, the quantities µ and η can be approached in terms of the lowest unoccupied molecular orbital (*LUMO*) and the highest occupied molecular orbital (*HOMO*) energy values, applying the finite difference approximation scheme, as µ ≈ (*E_LUMO_* + *E_HOMO_*)/2 and η ≈ (*E_LUMO_* *−* *E_HOMO_*)/2 ([27,28,29,30], and references therein). The relative global nucleophilicity index (*N*) of the reactive compounds, reflecting their global nucleophilic power, can be simply expressed in terms of their negative values of ionization potentials (*IPs*) or in terms of the negative *HOMO* energy values as a crude approximation of *IPs*, *i.e.*, the high (low) nucleophilicity of the compounds can be associated with their low (high) *IP* or *E_HOMO_* ([32,33,34], and references therein). In our study, the relative global *N* index values were obtained by means of the *HOMO* energy values, using the expression, *N* = *E_HOMO(Nu)_* *−* *E_HOMO(TCE)_* [32,33,34,35], where tetracyanoethylene (TCE), representing the lowest *HOMO* energy among a long series of organic molecules ([32], references therein), was used as reference. This definition leads to positive *N* index values. While the global electrophilic or nucleophilic indices reflect the extensive reactivity property of the molecules, their local reactivity indices, defined in terms of Fukui function (*FF*) values, can be used to predict which site of the molecule is most likely to be reactive. The power of the *FF* approach lies in the fact that the regioselectivity of the reactions can be assessed without a modeling the chemical reaction itself. *FF* can be usefully employed to identify the most probable reactive sites within a molecule, as well as to predict intramolecular reactivity, or to measure how the reactive site(s) of molecules can be affected by the introduction of electron-donating or -withdrawing group(s). In this work, the electrophilic (*F^+^_k_*) and nucleophilic (*F^−^_k_*) *FF* values, condensed to the particular k atoms of molecules, were assessed by means of the single point frontier molecular orbital (*FMO*) approach, applying the *LUMO* and the *HOMO* eigenvectors, respectively, and the matrix of the overlap integrals [36] (more computational details of *FF* values are given in the Experimental section). By contrast to most frequently used finite-difference approximation techniques, often leading to negative *FF* values, the single-point *FMO* approach yields the positive *FF* values ([36,37], and references therein).

**Figure 1:**
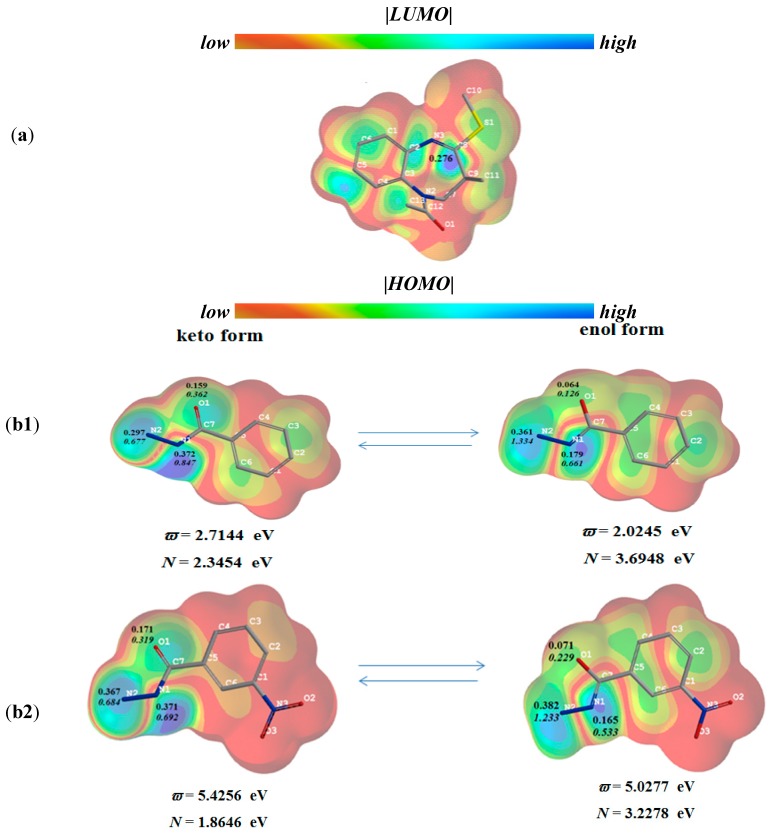
(**a**) |*LUMO*| map and electrophilic Fukui function (*F^+^_k_*) value of thioether compound **2d**; (**b1**) |*HOMO*| map and nucleophilic Fukui index (*F^−^_k_*) values of keto- and enol- form of benzohydrazide; (**b2**) |*HOMO*| map and *F^−^_k_* values of keto- and enol-form of 3-nitro-benzohydrazide. The global reactivity indices of the compounds: electrophilicity index (ϖ) and nucleophilicity index (N). |*LUMO*| and |*HOMO*| izo-values = 0.02.

**Scheme 2:**
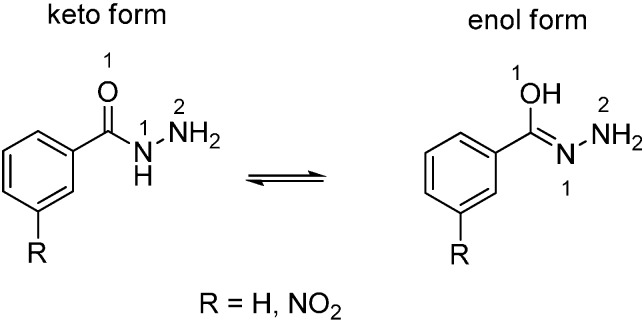
Keto-enol tautomeric equilibria for benzohydrazide molecules.

As shown in Figure 1a, the absolute *LUMO* density (|*LUMO*|) of thioether compound **2d** and the highest electrophilic *FF* value (*F^+^_k_*) is mainly condensed upon the carbon atom of the thioether molecule as the most probable electrophilic site, participating in the interaction with benzohydrazide or 3-nitrobenzohydrazide as nucleophiles (Scheme 1, synthesis pathway (ii) and resulting in the substitution of methylsulfanyl group by both benzohydrazides via the nucleophilic substitution reaction mechanism. As it has been described in literature [27], benzohydrazide molecules may undergo keto-enol tautomerism, which may determine their nucleophilic power (Scheme 2).

The calculations showed that the absolute *HOMO* density (|*HOMO*|) of the keto forms of benzohydrazide and 3-nitrobenzohydrazide as nucleophiles resides mainly upon their hydrazide moiety (Figure 1b1,b2); benzohydrazide (Figure 1b1) exhibits the lower global ϖ value and the higher global *N* value than those of 3-nitrobenzohydrazide (Figure 1b2). This implies that 3-nitro benzohydrazide possesses a lower propensity to act as a nucleophile in the reaction with thioether compound **2d** as an electrophile. In addition, the local nucleophilicity index (*N_k_*) values of the keto form of the nucleophile were assessed at O_1_, N_1_ and N_2_ atoms of the hydrazide moiety of both hydrazides. The *N_k_* values were obtained by multiplying global nucleophilic (*N*) and local nucleophilic (*F^−^_k_*) indices. The calculation yielded *N*_O1_ = 0.362 eV, *N*_N1_ = 0.847 eV and *N*_N2_ = 0.677 eV for benzohydrazide, and *N*_O1_ = 0.319 eV, *N*_N1_ = 0.692 eV and *N*_N2_ = 0.684 eV for 3-nitrobenzohydrazide. These data show that the highest local nucleophilic power of the compounds is condensed on the N_1_ atom of hydrazide moiety. Nevertheless, by contrast to the computation data, the experimental results suggested that the terminal N_2_ atom of hydrazides is the nucleophilic site interacting with the thioether compound **2d**. This enables us to propose that in these reactions, the enol form of benzohydrazide compound might be a proximate nucleophile. Note that the participation of the enol form of benzohydrazide, as a more reactive nucleophile compared to its keto form, has previously been proposed by Campodonico *et al.* [38], who examined the reactivity of benzohydrazides in acetylation reactions. Based upon DFT computational and experimental results, they suggested that benzohydrazide as a nucleophile, interacting with nitrophenyl acetate as an electrophile, may establish an intramolecular proton rearrangement, and the enol form of the benzohydrazide may be the active species for a nucleophilic attack. In our study, the assessment of the global ϖ and *N* index values of benzohydrazide and 3-nitro-benzohydrazide (Figure 1b1,b2) showed that the global nucleophilic powers of the enol forms of the compounds are markedly higher as compared with the nucleophilic powers of their keto forms. The local reactivity *N_k_* values at O_1_, N_1_ and N_2_ sites of enol forms were calculated as 0.599 eV, 0.661 eV and 1.335 eV, respectively, for benzohydrazide, and as 0.229 eV, 0.533 eV and 1.233 eV, respectively, for 3-nitrobenzohydrazide. These data show that the highest local nucleophilicity of the compounds is condensed on the terminal N_2_ atom of the hydrazide moiety which is expected to be the most probable nucleophilic site for a nucleophilic attack, and the presence of a nitro group in the benzene ring of benzohydrazide markedly lowers its global nucleophilic power, as well as decreases the nucleophilic power of the local sites of hydrazide moiety.

**Figure 2:**
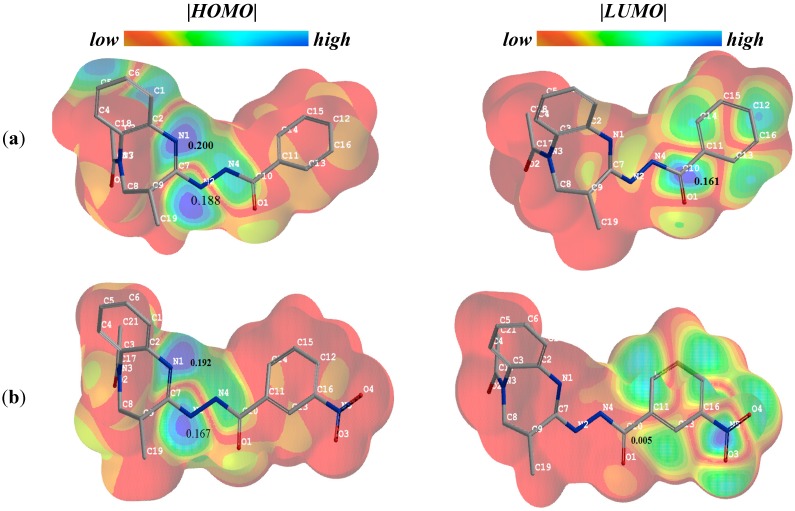
(**a**) The |*HOMO*| and |*LUMO*| maps of N’-(1-acetyl-2-methyl-2,3-dihydro-1H-1,5-benzodiazepin-4-yl)benzohydrazide and their highest nucleophilic (*F^−^_k_*) and electrophilic (*F^+^_k_*) Fukui function values; (**b**) **|***HOMO*| and |*LUMO*| maps of N'-(1-acetyl-2-methyl-2,3-dihydro-1*H*-1,5-benzodiazepin-4-yl)-3-nitrobenzohydrazide and their highest *F^−^_k_* and *F^+^_k_* values. |*HOMO*| and |*LUMO*| izo-values = 0.02.

Finally, we have attempted to tentatively rationalize the impact of the nitro group on the cyclization process of the intermediates *N*'-(1-acetyl-2-methyl-2,3-dihydro-1*H*-1,5-benzodiazepin-4-yl)benzo-hydrazide and *N*'-(1-acetyl-2-methyl-2,3-dihydro-1*H*-1,5-benzodiazepin-4-yl)-3-nitrobenzohydrazide into triazoles (Scheme 1, synthesis pathway iii). The |*HOMO*| and |*LUMO*| density maps of *N*'-(1,5-benzodiazepin-4-yl)benzohydrazide and *N*'-(1,5-benzodiazepin-4-yl)-3-nitro benzohydrazide as well as their *F^−^_k_* and *F^+^_k_* values for their nucleophilic and electrophilic reactive sites, respectively, are shown in Figure 2a,b.

The highest |*HOMO*| density and the highest *F^−^_k_* values of *N*'-(1,5-benzodiazepin-4-yl)benzohydrazide intermediate (Figure 2a) as well as of *N*'-(1,5-benzodiazepin-4-yl)-3-nitro benzohydrazide intermediate (Figure 2b) reside mainly on the N_1_ and N_2_ atoms of the diazepine and hydrazide fragments, respectively, as nucleophilic sites. The higher *F^−^_k_* values of the N_1_ atom of the diazepine fragment imply that this atom is the most plausible for the interaction with the carbonyl group of the hydrazide moiety. The |*LUMO*| density as well as the highest *F^+^_k_* value for *N*'-(1,5-benzodiazepin-4-yl)benzohydrazide (Figure 2a) are localized mainly on the carbon atom of the C=O group of the benzohydrazide as the most probable electrophilic site. Meanwhile, the obtained low |*LUMO*| density as well as low *F^+^_k_* function value for the carbon atom site of C=O group in *N*'-(1,5-benzodiazepin-4-yl)-3-nitro benzohydrazide (Figure 2b) clearly show that the presence of a nitro group in a benzene ring markedly lowers the local electrophilic power of the carbon site in a carbonyl group. This may be one of the main factors creating unfavorable conditions for a cyclization reaction. The synthesis of a broader set of triazole structure compounds, the assessment of their biological activity and analyses of quantitative structure-activity relationship (QSAR) of the compounds in order to define possible global and local reactivity descriptors of the compounds, responsible for their biological activity, will be the subject of our forthcoming works.

## 3. Experimental Section

### 3.1. General Information

Melting points were determinate on a Barnstead International MEL-TEMP capillary melting point apparatus and were not corrected. Elemental analyses (C, H, N, S) were performed on an Elemental Analyzer CE-440. IR spectra (4000–400 cm^−1^) were recorded on Perkin Elmer Spectrum GX FT-IR spectrometer in KBr pellets. ^1^H- and ^13^C-NMR spectra were recorded at 300 and 75 MHz, respectively, on an Varian Unity Inova 300 spectrometer with DMSO-d_6_ (compounds **3b**–**f**) and CDCl_3_ (compounds **2c**,**e**,**f** and **4a**–**f**) as solvent. The chemical shifts were referenced to tetramethylsilane (δ ^1^H = 0 ppm) and the solvent signal CDCl_3_ (δ ^13^C = 77.0 ppm) and DMSO-d_6_ (δ ^13^C = 39.5 ppm). The CH_3_, CH_2_, CH and C groups in ^13^C-NMR were differentiated by means of the APT or DEPT method. The reactions were monitored by TLC using TLC Silica gel 60 F_254_ (Merck) plates in systems: (a) benzene–methanol (v/v, 6:1) (compounds **2c**,**e**,**f**); (b) chloroform–ethyl acetate–methanol (v/v, 14:7:1,5) (compounds **3a**–**f**); (c) *n*-butanol–acetic acid–water (v/v, 4:2:1) (compounds **4a**–**f**). Visualization was made with UV light (254 nm) and with iodine vapor. Thiolactams **1a**–**f** were synthesized according to the described procedure [22,23,24]. 4-(Methylsulfanyl)-2,3-dihydro-1*H*-1,5-benzodiazepines **2a**,**b**,**d** were synthesized from thiolactams **1a**,**b**,**d** by treatment with methyl iodide [25,26].

#### 3.1.1. General Procedure for the Synthesis of 4-(Methylsulfanyl)-2,3-dihydro-1*H*-1,5-benzodiazepines **2c**,**e**,**f**

A mixture of the appropriate tetrahydro-1*H*-1,5-benzodiazepine-2-thione **1c**,**e**,**f** (0.01 mol) in benzene (50 mL), benzyltriethylammonium chloride (1.48 g, 0.0065 mol), methyl iodide (2.5 mL, 0.04 mol) and 40% NaOH (10 mL) was stirred at room temperature. After the completion of the reaction (5–8 h, by TLC monitoring) the filtrate was diluted with chloroform (100 mL) and water (100 mL). The organic layer was separated and aqueous phase extracted twice with 25 mL of chloroform. The combined organic phases were dried (Na_2_SO_4_) and evaporated to dryness in vacuum and the resulting thick oily residue was crystallized from ethanol to give the pure target compounds.

*1-Acetyl-3-methyl-4-(methylsulfanyl)-2,3-dihydro-1H-1,5-benzodiazepine* (**2c**). Yield 87%; m.p. 105–107 °C; IR ν: 1650 (C=O), 1598 (C=N) cm^−1^; ^1^H-NMR (CDCl_3_) δ: 1.20 (d, 3H, *J =* 6.9 Hz, CH_3_), 1.81 (s, 3H, CH_3_), 2.40 (s, 3H, CH_3_), 2.98 (m, 1H, CH), 3.54 (dd, 1H, *J =* 6.0 and 12.6 Hz, CH_2_), 4.68 (dd, 1H, *J =* 12.9 and 12.6 Hz, CH_2_), 7.08–7.39 (m, 4H, Ar-H) ppm; ^13^C-NMR (CDCl_3_) δ: 12.7 (3-CH_3_), 13.1 (4-CH_3_ ), 22.6 (1-CH_3_ ), 37.8 (C-3), 59.3 (C-2), 124.5 (CH), 124.6 (CH), 128.3 (CH), 128.9 (CH), 131.4 (C), 146.7 (C), 170.6 (CO), 177.7 (=C-S) ppm. Anal. Calcd. for C_13_H_16_N _2_OS (248.35): C 62.87, H 6.49, N 11.28, S 12.91. Found: C 62.92, H 6.47, N 11.30, S 12.87.

*3-Methyl-4-(methylsulfanyl)-N-phenyl-2,3-dihydro-1H-1,5-benzodiazepine-1-carboxamide* (**2e**). Yield 80%; m.p. 140–141 °C; IR ν: 3300 (NH), 1662 (C=O), 1596 (C=N) cm^−1^; ^1^H-NMR (CDCl_3_) δ: 1.22 (d, 3H, *J =* 6.7 Hz, CH_3_), 2.44 (s, 3H, S-CH_3_), 3.02 (m, 1H, CH), 3.79 (br.s, 1H, CH_2_), 4.43 (br.s, 1H, CH_2_), 6.18 (s, 1H, NH), 6.90–7.49 (m, 9H, Ar-H) ppm; ^13^C-NMR (CDCl_3_) δ: 12.9 (4-CH_3_), 13.4 (3-CH_3_), 38.3 (C-3), 59.8 (C-2), 119.0 (2 × CH), 122.9 (CH), 125.2 (CH), 125.7 (C), 128.3 (CH), 128.7 (2 × CH), 129.1 (CH), 129.9 (CH), 138.6 (C), 147.5 (C), 153.9 (CO), 178.4 (=C-S) ppm. Anal. Calcd. for C_18_H_19_N_3_OS (325.43): C 66.43, H 5.88, N 12.91, S 9.85. Found: C 66.57, H 5.79, N 12.98, S 9.81.

*2-Methyl-4-(methylsulfanyl)-N-phenyl-2,3-dihydro-1H-1,5-benzodiazepine-1-carboxamide* (**2f**). Yield 86%; m.p. 125–127 °C; IR ν: 3302 (NH), 1679 (C=O), 1592 (C=N) cm^−1^; ^1^H-NMR (CDCl_3_) δ: 1.29 (d, 3H, *J =* 6.3 Hz, CH_3_), 2.35–2.46 (m, 2H, CH_2_), 2.48 (s, 3H, S-CH_3_), 5.32 (m, 1H, CH), 6.06 (s, 1H, NH), 6.98–7.49 (m, 9H, Ar-H) ppm; ^13^C-NMR (CDCl_3_) δ: 13.2 (4-CH_3_ ), 19.4 (2-CH_3_), 41.3 (C-3), 59.71 (C-2), 118.9 (2 × CH), 122.7 (CH), 125.3 (CH), 125.6 (CH), 127.8 (C), 128.6 (2 × CH), 129.4 (CH), 130.1 (CH), 138.6 (C), 148.3 (C), 153.4 (CO), 173.6 (=C-S) ppm. Anal. Calcd. for C_18_H_19_N_3_OS (325.43): C 66.43, H 5.88, N 12.91, S 9.85. Found: C 66.51, H 5.81, N 12.96, S 9.77.

#### 3.1.2. General Procedure for the Synthesis of *N*'-(2,3-Dihydro-1*H*-1,5-benzodiazepin-4-yl)-3-nitro-benzohydrazides **3b**–**f**

A mixture of 3-nitrobenzohydrazide (2g, 0.011 mol) and 4-(methylsulfanyl)-2,3-dihydro-1*H*-1,5-benzodiazepine **2b**–**f** (0.007 mol) in dry ethanol (50 mL) was stirred at 45 °C. After the completion of the reaction (18–26 h, by TLC monitoring), the mixture was concentrated under reduced pressure to a volume of 30 mL. After cooling, the mixture was kept in a refrigerator overnight, and the precipitate was collected and recrystallized from an ethanol to give the pure compounds **3b**–**f**.

*N'-(1-Benzyl-2-methyl-2,3-dihydro-1H-1,5-benzodiazepin-4-yl)-3-nitrobenzohydrazide* (**3b**). Yield 71%; m.p. 169–171 °C; IR: ν 3180 (NH), 3120 (NH), 1670 (CO), 1594 (C=N), 1528 (NO_2_), 1347 (NO_2_) cm^−1^; ^1^H-NMR (DMSO-*d*_6_) δ: 1.05 (3H, d, *J =* 6.3Hz, CH_3_), 2.7-4.0 (3H, m, CH_2_CH), 4.30 and 4.43 (2H, AB-q, *J =* 14.8 Hz, CH_2_Ph), 6.95–8.90 (14H, m, Ar-H, NH), 10.54 (1H, s, NH) ppm; ^13^C-NMR (DMSO-*d*_6_) δ: 15.0 (2-CH_3_), 40.9 (C-3), 53.2 (5-CH_2_), 59.5 (C-4), 122.1 (CH), 123.0 (CH), 123.5 (CH), 126.7 (CH), 126.9 (CH), 127.7 (2 × CH), 128.0 (C), 128.1 (2 × CH), 129.4, 129.6, 134.2, 134.4, 134.5, 135.6 (C), 138.5(C), 147.6 (C-NO_2_), 155.9 (C=N), 163.7 (2-CO) ppm. Anal. Calcd. for C_24_H_23_N_5_O_3_ (429.47): C 67.12, H 5.40, N 16.31. Found: C 67.24, H 5.31, N 16.23.

*N'-(1-Acetyl-3-methyl-2,3-dihydro-1H-1,5-benzodiazepin-4-yl)-3-nitrobenzohydrazide* (**3c**). Yield 85%; m.p. 245–247 °C; IR: 3205 (NH), 1669 (CO), 1600 (C=N), 1526 (NO_2_), 1350 (NO_2_) cm^−1^; ^1^H-NMR (DMSO-*d*_6_) δ: 1.44 (3H, d, *J =* 6.7 Hz, CH_3_), 1.84 (3H, s, CH_3_), 2.96 (1H, m, CH), 3.61 (1H, dd, *J =* 6.4 and *J =* 12.7 Hz, CH_2_), 4.38 (1H, dd, *J =* 12.7 and 12.9 Hz, CH_2_), 7.12– 8.40 (8H, m, Ar-H), 8.85 (1H, s, NH), 10.55 (1H, s, NH) ppm; ^13^C-NMR (DMSO-d_6_) δ: 13.1 (3-CH_3_), 22.6 (1-CH_3_), 28.3 (C-3), 54.4 (C-2), 123.0 (CH), 124.6 (CH), 126.1 (CH), 127.9 (CH), 129.8 (CH), 130.2 (CH), 130.8 (CH), 131.2 (C), 131.4 (C), 134.3 (CH), 134.9 (C), 147.7 (C-NO_2_ ), 150.4 (C=N), 156.3 (CO), 169.2 (1-CO) ppm. Anal. Calcd. for C_19_H_19_N_5_O_4_ (381.39): C 59.84, H 5.02, N 18.36. Found: C 59.93, H 4.97, N 18.21.

*N'-(1-Acetyl-2-methyl-2,3-dihydro-1H-1,5-benzodiazepin-4-yl)-3-nitrobenzohydrazide* (**3d**). Yield 65%; m.p. 249–251 °C; IR ν: 3206 (NH), 1669 (CO), 1587 (C=N), 1528 (NO_2_), 1349 (NO_2_) cm^−1^; ^1^H-NMR (DMSO-*d*_6_) δ: 1.11 (3H, d, ^3^*J =* 6.6 Hz, CH_3_), 1.64 (3H, s, CH_3_), 2.59 (1H, m, CH_2_), 5.01 (1H, m, CH), 7.17–8.76 (8H, m, Ar-H), 8.98 (1H, br.s, NH), 10.59 (1H, br.s, NH) ppm; ^13^C-NMR (DMSO-*d*_6_) δ: 18.7 (2-CH_3_), 22.7 (1-CH_3_), 37.2 (C-3), 53.3 (C-2), 122.0 (CH), 122.3 (CH), 124.5 (CH), 124.9 (CH), 125.6 (CH), 129.2 (CH), 129.7 (CH), 130.6 (C), 131.0 (C), 134.1 (CH), 137.9 (C), 147.6 (C-NO_2_), 153.6 (C=N), 160.8 (CO), 168.7 (1-CO) ppm. Anal. Calcd. for C_19_H_19_N_5_O_4_ (381.39): C 59.84, H 5.02, N 18.36. Found: C 60.01, H 4.94, N 18.28.

*3-Methyl-4-[2-(3-nitrobenzoyl)hydrazino]-N-phenyl-2,3-dihydro-1H-1,5-benzodiazepine-1-carboxamide* (**3e**). Yield 62%; m.p. 230–232 °C; IR ν: 3302 (NH), 3071 (NH), 1681 (CO), 1594 (C=N), 1530 (NO_2_), 1352 (NO_2_) cm^−1^; ^1^H-NMR (DMSO-*d*_6_) δ: 1.45 (3H, br.d, CH_3_), 2.92 (1H, m, CH), 3.83 (1H, br.s, CH_2_), 4.19 (1H, br.s, CH_2_), 6.92–8.72 (16H, m, 3NH, Ar-H) ppm; ^13^C-NMR (DMSO-d_6_) δ: 13.1 (3-CH_3_), 28.9 (C-3), 56.5 (C-2), 121.1 (2 × CH), 122.9 (CH), 123.2 (CH), 124.6 (CH), 126.0 (CH), 128.1 (CH), 128.3 (2 × CH), 129.2 (CH), 130.4 (CH), 130.5 (CH), 131.4 (C), 132.7 (C), 134.5 (CH), 135.1 (C), 139.3 (C-NH), 148.0 (C-NO_2_), 150.6 (C), 155.0 (CO), 156.5 (CO) ppm. Anal. Calcd. for C_24_H_22_N _6_O_4_ (458.47): C 62.87, H 4.84, N 18.33. Found: C 62.91, H 4.77, N 18.25.

*2-Methyl-4-[2-(3-nitrobenzoyl)hydrazino]-N-phenyl-2,3-dihydro-1H-1,5-benzodiazepine-1-carboxamide* (**3f**). Yield 59%; m.p. 197–200 °C; IR ν: 3424 (NH), 3063 (NH), 1684 (CO), 1594 (C=N), 1531 (NO_2_), 1349 (NO_2_) cm^−1^; ^1^H-NMR (DMSO-*d*_6_) δ: 1.20 (3H, d, *J =* 6.1Hz, CH_3_), 1.99–2.75 (2H, m, CH_2_), 4.86 (1H, m, CH), 6.8–8.8 (14H, m, NH, Ar-H), 8.96 (1H, s, NH), 10.64 (1H, s, NH) ppm; ^13^C-NMR (DMSO-*d*_6_) δ: 19.1 (2-CH_3_), 37.6 (C-3), 54.5 (C-2), 120.1 (2 × CH), 122.3 (CH), 123.1 (CH), 124.7 (CH), 124.8 (CH), 125.6 (CH), 128.2 (2 × CH), 128.8 (CH), 129.1 (CH), 129.8 (CH), 131.1 (C), 132.6 (C), 134.1 (CH), 138.5 (C), 139.6 (C-NH), 147.6 (C-NO_2_), 153.6 (C=N), 158.7 (CO), 161.1 (CO) ppm. Anal. Calcd. for C_24_H_22_N _6_O_4_ (458.47): C 62.87, H 4.84, N 18.33. Found: C 62.94, H 4.81, N 18.23.

#### 3.1.3. *Synthesis of 6-Benzyl-4-methyl-1-(3-nitrophenyl-5,6-dihydro-4H-[1,2,4]triazolo[4,3-a][1,5] benzodiazepine* (**4a**)

The mixture of 3-nitrobenzohydrazide (2 g, 0.011 mol) and 4-(methylsulfanyl)-2,3-dihydro-1*H*-1,5-benzodiazepine (**2a**, 2.08g. 0.007 mol) in dry ethanol (50 mL) was stirred at 45 °C temperature. After the completion of the reaction (18 h, by TLC monitoring) the mixture was filtered, a solid residue was dissolved in 50 mL of butanol-1 and was heated 9 h, under reflux. The solution was concentrated under reduced pressure to a volume of 20 mL. After cooling the precipitate was collected and recrystallized from ethyl acetate to give the pure compound **4a**. Yield 71%; m.p. 188–189 °C; IR ν: 1602 (C=N), 1541 (NO_2_), 1348 (NO_2_) cm^−1^; ^1^H-NMR (CDCl_3_) δ: 1.54 (3H, d, *J =* 6.6Hz, CH_3_), 3.04 (1H, m, CH), 3.3–3.6 (2H, m, CH_2_), 4.23 and 4.52 (2H, AB-q, *J =* 14.2 Hz, CH_2_), 6.75–7.3 (9H, m, Ar-H), 7.51 (1H, m, H-5'), 7.72 (1H, m, H-6'), 8.25 (1H, m, H-4'), 8.32 (1H, m, H-2') ppm; ^13^C-NMR (CDCl_3_) δ: 13.3 (4-CH_3_), 29.4 (C-4), 57.0 (6-CH_2_), 65.0 (C-5), 123.0 (CH), 123.4 (CH), 123.5 (CH), 124.3 (CH), 125.1 (CH), 127.4 (CH), 127.8 (2 × CH), 128.5 (2 × CH), 128.8 (C), 129.3 (C), 129.6 (CH), 129.7 (CH), 134.2 (CH), 137.3 (C), 142.7 (C), 148.2 (C-NO_2_), 150.6 (C), 157.9 (C) ppm. Anal. Calcd for C_24_H_21_N_5_O_2_ (411.46): C 70.06, H 5.14, N 17.02. Found: C 70.14, H 5.03, N 16.91.

#### 3.1.4. General Procedure for the Synthesis of 1-(3-Nitrophenyl)-5,6-dihydro-4*H*-[1,2,4]triazolo[4,3-*a*][1,5]benzodiazepines **4b**–**f**

A suspension of *N*'-(2,3-dihydro-1*H*-1,5-benzodiazepin-4-yl)-3-nitrobenzohydrazide (0.007 mol) **3b**–**f** in 1-butanol (50 mL) was heated under reflux. After the completion of the reaction (9–21 h, by TLC monitoring) the solution was concentrated under reduced pressure to a volume of 20 mL. After cooling the precipitate was collected and recrystallized from an ethyl acetate to give the pure compounds **4b**–**f**.

*6-Benzyl-5-methyl-1-(3-nitrophenyl-5,6-dihydro-4H-[1,2,4]triazolo[4,3-a][1,5]benzodiazepine* (**4b**). Yield 74%; m.p. 181–182 °C; IR ν: 1599 (C=N), 1541 (NO_2_), 1349 (NO_2_) cm^−1^. ^1^H-NMR (CDCl_3_) δ: 1.27 (3H, br.d, CH_3_), 2.41 (1H, br. s, CH_2_), 3.42 (1H, br.s, CH_2_), 4.06 (1H, m, CH), 4.23 and 4.54 (2H, br. AB-q, CH_2_), 6.75–7.60 (11H, m, Ar-H), 8.22 (1H, m, H-4'), 8.30 (1H, m, H-2') ppm; ^13^C-NMR (CDCl_3_) δ: 15.6 (5- CH_3_), 31.3 (C-4), 54.5 (6-CH_2_), 62.1 (C-5), 123.2 (CH), 124.0 (CH), 124.1 (CH), 124.7 (CH), 126.2 (C), 127.2 (CH), 127.8 (2 × CH), 128.3 (2 × CH), 128.6 (CH), 129.2 (CH), 129.4 (CH), 130.8 (C), 133.9 (CH), 137.7 (C), 139.5 (C), 148.1 (C-NO_2_), 150.2 (C), 154.9 (C) ppm. Anal. Calcd. for C_24_H_21_N_5_O_2_ (411.46): C 70.06, H 5.14, N 17.02. Found: C 70.11, H 5.08, N 16.94.

*6-Acetyl-4-methyl-1-(3-nitrophenyl-5,6-dihydro-4H-[1,2,4]triazolo[4,3-a][1,5]benzodiazepine* (**4c**). Yield 78%; m.p. 247–248 °C; IR ν: 1668 (CO), 1600 (C=N), 1539 (NO_2_), 1353 (NO_2_) cm^−1^; ^1^H-NMR (CDCl_3_) δ: 1.64 (3H, d, *J =* 6.7 Hz, CH_3_), 1.97 (3H, s, CH_3_), 2.97 (1H, m_,_ CH), 3.66 (1H, dd, *J =* 12.7 and 6.3 Hz, CH_2_), 4.63 (1H, dd, *J =* 12.7 and 12.9 Hz, CH_2_), 6.94-8.33 (8H, m, Ar-H) ppm; ^13^C-NMR (CDCl_3_) δ: 13.1 (4-CH_3_), 22.8 (6-CH_3_), 28.9 (C-4), 55.2 (C-5), 123.0 (CH), 124.7 (CH), 125.9 (CH), 128.1 (C), 129.8 (CH), 130.3 (2 × CH), 130.9 (CH), 131.9 (C), 134.0 (CH), 135.3 (C), 148.1 (C-NO_2_), 150.6 (C), 156.7 (C), 169.8 (6-CO) ppm. Anal. Calcd. for C_19_H_17_N_5_O_2_ (363.37): C 62.80, H 4.72, N 19.27. Found: C 62.71, H 4.68, N 19.16.

*6-Acetyl-5-methyl-1-(3-nitrophenyl-5,6-dihydro-4H-[1,2,4]triazolo[4,3-a][1,5]benzodiazepine* (**4d**). Yield 85%; m.p. 250–252 °C; IR ν: 1657 (CO), 1600 (C=N), 1534 (NO_2_), 1354 (NO_2_) cm^−1^; ^1^H-NMR (CDCl_3_) δ: (two rotomer’s in ratio 90:10, in brackets—smaller) 1.32 (3H, d, *J =* 6.4Hz, CH_3_), [1.43 (3H d, *J* = 6.4 Hz, CH3)], 1.89 (3H, s, CH_3_), [2.17 (3H, s, CH3)], 2.33 (1H, dd, *J =* 12.4 and 15.0 Hz, CH_2_), [2.39 (1H, dd, *J* = 11.4 and 15.0 Hz, CH2)], 3.44 (1H, dd, *J =* 6.0 and 15.0 Hz, CH_2_), [3.49 (1H, dd, *J* = 6.0 and 14.9 Hz, CH2)], [4.67 (1H, m, CH)], 5.30 (1H, m, CH), 6.90–8.66 (8H, m, Ar-H) ppm; ^13^C-NMR (CDCl_3_) δ: 19.1 (5-CH_3_), 23.2 (6-CH_3_), 30.0 (C-4), 54.7 (C-5), 122.9 (CH), 124.7 (CH), 125.7 (CH), 128.1 (C), 130.0 (CH), 130.2 (CH), 130.3 (CH), 132.4 (CH), 133.2 (C), 133.8 (CH), 134.3 (C), 148.1 (C-NO_2_), 150.2 (C), 153.9 (C), 169.1 (6-CO) ppm. Anal. Calcd. for C_19_H_17_N_5_O_2_ (363.37): C 62.80, H 4.72, N 19.27. Found: C 62.81, H 4.63, N 19.19.

*6-Carbamoyl-4-methyl-1-(3-nitrophenyl-5,6-dihydro-4H-[1,2,4]triazolo[4,3-a][1,5]benzodiazepine* (**4e**). Yield 73%; m.p. 239–241 °C; IR ν: 3423 (NH), 1681 (CO), 1594 (C=N), 1529 (NO_2_), 1349 (NO_2_) cm^−1^; ^1^H-NMR (CDCl_3_) δ: 1.64 (3H, br.d, CH_3_), 2.97 (1H, m, CH), 3.81, (1H, m, CH_2_ ), 4.55 (1H, m, CH_2_), 6.17 (1H, s, NH), 6.96–8.28 (13H, m, Ar-H) ppm; ^13^C-NMR (CDCl_3_) δ: 13.1 (4-CH_3_), 29.4 (C-4), 55.8 (C-5), 119.5 (2 × CH), 123.0 (CH), 123.8 (CH), 124.7 (CH), 126.5 (CH), 128.1 (C), 129.0 (2 × CH), 130.0 (CH), 130.4 (CH), 130.8 (CH), 131.1 (CH), 133.2 (C), 134.1 (C), 134.5 (CH), 137.8 (C), 147.9 (C-NO_2_), 150.7 (C), 153.4 (C), 156.8 (6-CO) ppm. Anal. Calcd. for C_24_H_20_N_6_O_3_ (440.45): C 65.45, H 4.58, N 19.08. Found: C 65.53, H 4.62, N 18.93.

*6-Carbamoyl-5-methyl-1-(3-nitrophenyl-5,6-dihydro-4H-[1,2,4]triazolo[4,3-a][1,5]benzodiazepine* (**4f**). Yield 65%; m.p. 222–223 °C; IR ν: 3411 (NH), 1678 (CO), 1595 (C=N), 1533 (NO_2_), 1350 (NO_2_) cm^−1^. ^1^H-NMR (CDCl_3_) δ: 1.42 (3H, d, *J =* 6.3 Hz, CH_3_), 2.37 (1H, dd, *J =* 11.6 and 14.8 Hz, CH_2_), 3.51, (1H, m, *J =* 6.1 and 14.9 Hz, CH_2_), 5.32 (1H, m, CH), 6.19 (1H, s, NH), 7.00–8.30 (13H, m, Ar-H) ppm; ^13^C-NMR (CDCl_3_) δ: 19.8 (5-CH_3_), 30.3 (C-4), 55.2 (C-5), 119.5 (2 × CH), 122.8 (CH), 123.6 (CH), 124.3 (CH), 126.0 (CH), 127.9 (C), 128.8 (2 × CH), 130.3 (2 × CH), 130.6 (CH), 131.5 (C), 132.7 (CH), 133.5 (C), 134.2 (CH), 137.8 (C), 147.8 (C-NO_2_), 150.2 (C), 152.7 (C), 153.9 (6-CO) ppm. Anal. Calcd. for C_24_H_20_N_6_O_3_ (440.45): C 65.45, H 4.58, N 19.08. Found: C 65.59, H 4.60; N 18.96.

### 3.2. Quantum Mechanical Computations

#### 3.2.1. General

The quantum mechanical computation (QMC) was performed using the Spartan 10 software package [39]. The initial refinement of the geometry of the compounds was carried out by the mechanical force field (MMFF94) method, and further optimization was performed using semi-empirical PM6 Hamiltonian. The final optimization and computation of the compounds were performed by DFT-B3LYP method in conjunction with 6-31G* basis set. All structures were globally optimized without symmetry restrictions, and the stationary points of the optimized structures were verified by frequency analysis. For all the optimized structures, no imaginary frequencies were defined.

#### 3.2.2. The Assessment of DFT-Based Global and Local Reactivity Indices

The DFT quantities such as the global electronic chemical potential index (µ), the global chemical hardness index (η) and the global electrophilicity index (ϖ) of molecules were assessed within a framework of Koopman’s theory approach. The global nucleophilicity index (*N*) values of the nucleophilic compounds were obtained by applying the method proposed by Domingo *et al.* [35].

For prediction of the local reactivity of the compounds, the Fukui function (*FF*) values for electrophilic attack (*F^−^_k_*) and nucleophilic attack (*F^+^_k_*) were assessed by the single-point frontier molecular orbital (*FMO*) approach proposed by Contreras *et al.* [36], involving the highest unoccupied molecular orbital (*HOMO*) and the lowest unoccupied molecular orbital (*LUMO*) coefficients (eigenvectors), respectively, and the matrix of the overlap integrals between the basis vectors. The calculations were performed as follows: first, the FF values for the orbital components (α) of the atomic orbital (µ) (*F*^α^_µ_) were computed as $F_{\text{μ}}^{\alpha }=\sum_{\text{ν}}c_{\text{μα}}c_{\text{να}}S_{\text{μα}}=c_{\text{μα}}^{2}+c_{\text{μα}}\sum_{\nu \neq \alpha }c_{\text{να}}S_{\text{μν}}$, where *C*_µα_ and *C*_να_ are µ-th coefficient and ν-th expansion coefficient, respectively, and S_µν_ is an overlap integral between the µ and ν orbital components; next, the *FF* values, condensed upon k atom of the compound (*F^±^_k_*), were obtained by summing *F*^α^_µ_ values ($F_{k}^{\pm }=\sum_{\mu ∍k}F_{\text{μ}}^{\alpha }$) and finally checked for the normalization condition ($\sum_{k}F_{k}^{\pm }=1$). These calculations were performed by employing PYTHON open-source programming language. The local *N_k_* values condensed upon particular reactive sites of nucleophiles were obtained by multiplying the global *N* index value by local *F^−^_k_* values, *N_k_ = N × F^−^_k_*.

## 4. Conclusions

The interaction of the 4-methylsulfanyl-1,5-benzodiazepine thioethers **2a**–**f** with 3-nitro-benzo- hydrazide afforded the corresponding 1,5-benzodiazepin-2-ylidene)-3-nitrobenzohydrazides **3a**–**f**. The thermal cyclization of **3a**–**f** provided a simple way to obtain novel tricyclic 5,6-dihydro-4*H*-[1,2,4]triazolo[4,3-*a*][1,5] benzodiazepine derivatives **4a**–**f**. These reactions were found to proceed at a higher temperature and much more slowly as compared with those when benzohydrazide was used instead of 3-nitrobenzohydrazide. To tentatively rationalize the experimental results, a preliminary computational analysis of the compounds involved in the reaction pathway was performed within the framework of the density functional theory (DFT) approach. The assessment of the DFT-based global and local reactivity indices of the compounds clearly showed that the presence of a nitro group on the benzene ring of the benzohydrazide markedly lowers the global nucleophilicity of the compound as well as decreases the local nucleophilic power of its hydrazide moiety. Furthermore, the presence of a nitro group on the benzene ring of the 1,5-benzodiazepin-2-ylidene-3-nitrobenzohydrazide intermediate was found to markedly decrease the local electrophilic power of the carbon site in the carbonyl group. This may be one of the main factors creating unfavorable conditions for a cyclization reaction. Since triazole structure compounds are known to constitute a class of widely used antifungal agents, the synthesis of a broader set of triazoles, the assessment of their biological activity and the QSAR analyses of possible dependences of the biological activity of triazoles on their quantum mechanically assessed reactivity indices will be the subject of our forthcoming study.
